# Influence of age on nicotinic cholinergic regulation of blood flow in rat’s olfactory bulb and neocortex

**DOI:** 10.1186/s12576-024-00913-8

**Published:** 2024-03-15

**Authors:** Sae Uchida, Fusako Kagitani

**Affiliations:** Department of Autonomic Neuroscience, Tokyo Metropolitan Institute for Geriatrics and Gerontology, 35-2 Sakaecho, Itabashi-ku, Tokyo, 173-0015 Japan

**Keywords:** Olfactory bulb, Neocortex, Cerebral blood flow, Nicotinic acetylcholine receptor, Rat

## Abstract

The olfactory bulb receives cholinergic basal forebrain inputs as does the neocortex. With a focus on nicotinic acetylcholine receptors (nAChRs), this review article provides an overview and discussion of the following findings: (1) the nAChRs-mediated regulation of regional blood flow in the neocortex and olfactory bulb, (2) the nAChR subtypes that mediate their responses, and (3) their activity in old rats. The activation of the α4β2-like subtype of nAChRs produces vasodilation in the neocortex, and potentiates olfactory bulb vasodilation induced by olfactory stimulation. The nAChR activity producing neocortical vasodilation was similarly maintained in 2-year-old rats as in adult rats, but was clearly reduced in 3-year-old rats. In contrast, nAChR activity in the olfactory bulb was reduced already in 2-year-old rats. Thus, age-related impairment of α4β2-like nAChR function may occur earlier in the olfactory bulb than in the neocortex. Given the findings, the vasodilation induced by α4β2-like nAChR activation may be beneficial for neuroprotection in the neocortex and the olfactory bulb.

## Introduction

As background information for this review article, a brief history and scientific knowledge of basic and clinical research on the basal forebrain cholinergic system will be provided. In humans, the olfactory function starts declining after 65 years of age [1]. In addition to age-related impairment, olfactory decline is an early symptom of Alzheimer’s disease (AD) that appears before cognitive decline [2, 3]. The olfactory bulb, which is the first processing station of olfactory information in the brain, receives cholinergic basal forebrain input, as do the neocortex and hippocampus, which contribute to cognition and memory, respectively [4]. More specifically, magnocellular neurons in the basal forebrain provide widespread cholinergic innervation to the neocortex, hippocampus, and olfactory bulb. The bilateral horizontal and caudal portions of the cholinergic basal forebrain neurons, which are located in the substantia innominata (SI) and nucleus basalis of Meynert (NBM), project their axons to the neocortex. In turn, fibers of neurons located in the nucleus of the horizontal limb of the diagonal band of Broca (HDB) project to the olfactory bulb. The most rostral level of the cholinergic neurons of the basal forebrain, which is located in the medial septal nucleus (MS) and the nucleus of the vertical limb of the diagonal band of Broca (VDB), project mainly to the hippocampus [4, 5]. These cholinergic neurons of the basal forebrain undergo selective degeneration in patients with AD [6, 7]. Moreover, individual variations in cholinergic cell loss, from moderate to severe, are correlated with the degree of cognitive deterioration in these patients [7]. This indicates the importance of this cholinergic system for cognitive function. In vivo brain imaging studies have revealed a moderate structural decline in the gray matter volume of the basal forebrain cholinergic system during the adult life span, which worsens with advanced age [8]. A further decrease in the volume of the basal forebrain cholinergic system beyond the age effect alone were detected in early stages of AD [8, 9].

Presumed cholinergic terminations onto cerebral blood vessels from the magnocellular basal nucleus, in addition to interneuronal contacts, are evident in rodent studies that used the anterograde axonal-tracing technique [10]. The activation of cholinergic fibers originating in the NBM produces vasodilation, which leads to increases in regional blood flow in the neocortex in anesthetized rats [11–13]. Acetylcholine receptors consist of muscarinic and nicotinic receptors. The activation of not only muscarinic but also nicotinic receptors within the parenchyma of the neocortex is involved in the NBM cholinergic vasodilative system [11].

In the human neocortex, nicotinic acetylcholine receptors (nAChRs) exhibit a greater decline than do muscarinic acetylcholine receptors during the normal aging process as well as in patients with AD [14, 15]. Therefore, to understand the mechanisms of olfactory decline associated with cognitive decline in older adults and patients with AD, it is important to elucidate the regulation of regional blood flow mediated by the nicotinic cholinergic system in the neocortex and olfactory bulb, together with its aging process.

In this article, we focus on nAChRs, review results reported mainly by our research group using anesthetized rats and discuss on (1) nAChRs-mediated regulation of regional blood flow in the neocortex and the olfactory bulb, (2) nAChR subtype mediating their responses, and (3) their activity in old rats.

## Nicotinic cholinergic regulation of regional blood flow in the neocortex

### Nicotine injection

Nicotine, a nAChR agonist, has been demonstrated to increase regional cerebral blood flow when injected intravenously, especially in the neocortex, independent of mean arterial pressure [16–18]. In our investigation, we measured the blood flow in the frontal cortex by laser Doppler flowmetry in urethane-anesthetized artificially ventilated rats, before and after intravenous bolus injection of nicotine [18]. Nicotine at doses of 3–30 µg/kg increased neocortical blood flow in a dose-dependent manner, without significant changes in mean arterial pressure. At 300 µg/kg, nicotine increased neocortical blood flow in parallel with a marked increase in arterial pressure. The rapid increase in neocortical blood flow after the injection of 300 µg/kg of nicotine appears to be due to a passive increase in neocortical blood flow in response to the increased arterial pressure. Accordingly, nicotine doses of 30 μg/kg or less increased the neocortical blood flow without affecting systemic blood pressure is due to active dilation of neocortical vessels. Nicotine at the same doses had a similar effect to the parietal cortex in our animals.

The finding mentioned above is in line with evidence that the activation of nAChRs is involved in vasodilation in the neocortex by excitation of intracranial cholinergic fibers originating in the NBM of the basal forebrain projecting to the neocortex [11–13, 19].

### nAChR subtype

The increase in neocortical blood flow induced by intravenous injection of nicotine (30 μg/kg) is due to an activation of nAChRs in the brain; in fact, the response was not influenced by a nAChR antagonist (hexamethonium) which cannot transverse the blood–brain barrier, but was abolished by a nAChR antagonist (mechamylamine) that can cross it [18]. Activations of nAChRs in both the NBM and the neocortex are possibly involved in the nicotine-induced neocortical vasodilation [17, 18]. Furthermore, nitric oxide is necessary for this nicotine-induced increase in neocortical blood flow [20].

Of the various subtypes of nAChRs, the α4β2 and α7 subtypes are the most abundant and widespread in the mammalian brain, including the neocortex and NBM [21–23]. The increase in neocortical blood flow induced by nicotine was not influenced by methyllycaconitine, an α7-selective nAChR antagonist but was completely abolished by dihydro-β-erythroidine, an α4β2-preferring nAChR antagonist [24]. These results suggest that activation of α4β2-like nAChRs but not of α7 nAChRs in the NBM and the neocortex is responsible for the nicotine-induced neocortical vasodilation. However, although dihydro-β-erythroidine is often used as an α4β2-preferring nAChR antagonist [25, 26], we cannot exclude the possible contribution of other heterometric nAChRs, since dihydro-β-erythroidine can bind to heterometric neuronal nAChRs containing not only the α4β2 subtype but also the α4β4 [27], α3β2 [28], and α2β2 [29] subtypes.

### Aging

**Fig. 1:**
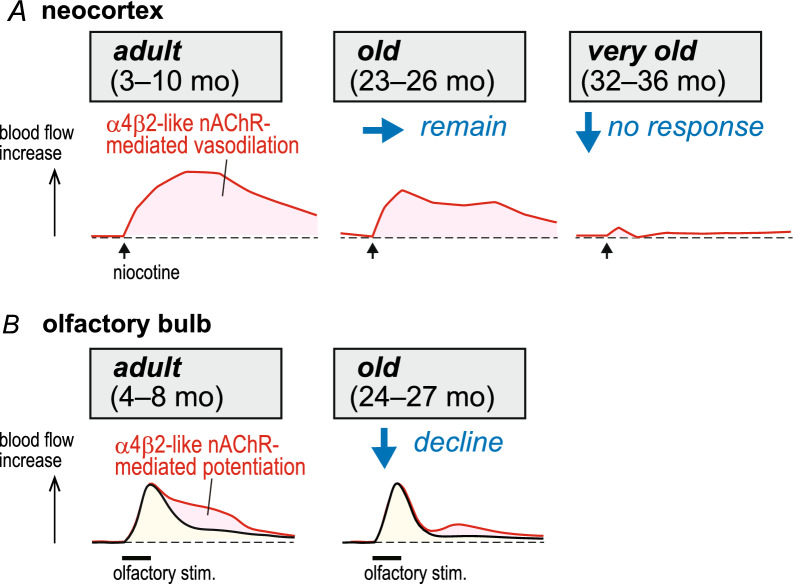
Schematic diagram showing the aging effects of α4β2-like nAChR-mediated neocortical vasodilation (**A**) and potentiation of vasodilation in the olfactory bulb (**B**). **A** Neocortical vasodilation induced by intravenous bolus injection of nicotine at a dose of 30 μg/kg was schematically illustrated [18]. **B** Vasodilation of the olfactory bulb induced by olfactory nerve stimulation before (black line) and after (red line) intravenous injection of nicotine at a dose of 30 μg/kg was schematically illustrated [54]. *nAChR* nicotinic acetylcholine receptor

The above-mentioned experiments showing an increase in neocortical blood flow induced by intravenous injection of nicotine (30 µg/kg) were performed in adult rats aged 3–10 months. Then we moved on to older animals [18, 30, 31]. In rats 23–26 months old (approximately 2 years old), a bolus injection of 30 µg/kg of nicotine increased neocortical blood flow to a similar extent as in the younger rats. A lower nicotine dose (3 µg/kg), however, was ineffective. In other words, the intensity of the response to nicotine remained unchanged in old rats, but the threshold became higher. In contrast, in 32–36 months old rats (approximately 3 years old), nicotine, at 3 or 30 µg/kg, had no significant effect on the neocortical blood flow (Fig. 1A). The decrease in the neocortical blood flow response in the very old animals is probably due to a decline in the number of nAChRs in the neocortex, as previously observed in both rodents and humans [14, 32].

## Nicotinic cholinergic regulation of regional blood flow in the olfactory bulb

### Nicotine injection

The effect of an intravenous bolus injection of nicotine on the blood flow in the olfactory bulb was investigated [33]. Nicotine at a dose of 30 µg/kg, increased neocortical blood flow [18], but did not increase blood flow in the olfactory bulb [33]. This result agrees with the fact that activation of the HDB in the basal forebrain, which is the main source of cholinergic input to the olfactory bulb [5, 34, 35], increased extracellular acetylcholine release in the olfactory bulb but failed to affect the blood flow in the olfactory bulb [36]. Our results suggest a functional difference between the olfactory bulb and neocortex regarding cerebral blood flow regulation through cholinergic activation.

Multiple in vivo studies in rodents have described that natural olfactory stimulation increases regional blood flow in the olfactory bulb, in association with neuronal activities [37–40]. The vasodilation in the olfactory bulb induced by olfactory stimulation is due to neurovascular coupling mechanisms [41, 42].

Next, we investigated the effect of nAChR activation by nicotine injection on the blood flow response in the olfactory bulb induced by olfactory stimulation in urethane-anesthetized artificially ventilated rats [43]. Odor stimulation (5% amyl acetate, 30 s) produced an increase in olfactory bulb blood flow without changes in frontal cortical blood flow or mean arterial pressure. An intravenous injection of nicotine at a dose of 30 µg/kg potentiated the odor-induced increased olfactory bulb blood flow, without changing the basal blood flow level.

The olfactory nerve transmits smell information from the olfactory epithelium to the olfactory bulb. Rodent studies indicated that the olfactory nerve increases its firing frequency depending on the odor concentration [44–46]. By varying the stimulus frequencies of olfactory nerve electrical stimulation, we could quantify the strength of odor stimulation. Our experiments demonstrated that unilateral, electrical stimulation of olfactory nerve produced current (≥ 100 μA) and frequency-dependent (≥ 5 Hz) increases in blood flow in the olfactory bulb ipsilateral to the stimulus without changes in frontal cortical blood flow or mean arterial pressure [47]. Furthermore, we observed that an intravenous injection of nicotine (30 μg/kg) augmented the olfactory bulb blood flow response to nerve stimulation. Nicotine-induced potentiation of olfactory bulb blood flow responses occurred with olfactory nerve stimulation at 2 and 20 Hz but not at 100 Hz [47]. This finding provides additional evidence that nAChR activation potentiates olfactory bulb blood flow responses to olfactory input, this potentiation occurs for intermediate or weak, but not strong, input. In a Ca^2+^ imaging study in the mouse olfactory bulb, Bendahmane et al. [48] indicated that electrical stimulation of the HDB leads to the activity-dependent modulation of glomerular odor responses, whereby weak-to-moderate responses are enhanced and strong responses are reduced. Thus, our results [47], almost agree with those of Bendahmane et al. [48].

### nAChR subtype

The above-mentioned nicotine-induced potentiation of olfactory bulb blood flow response to odor was negated by dihydro-β-erythroidine, an α4β2-preferring nAChR antagonist [43]. Thus, our results suggest that the activation of α4β2-like neuronal nAChRs in the brain potentiates olfactory sensory processing in the olfactory bulb. However, a contribution of not only α4β2-like nAChRs [49] but also α2- [50] and β4-containing nAChRs [51] in the olfactory bulb should be considered, since dihydro-β-erythroidine can bind to heterometric neuronal nAChRs other than the α4β2 subtype.

As described in “nAChR subtype” section, activation of α4β2-like nAChRs in the NBM and the neocortex is suggested to be responsible for the nicotine-induced neocortical vasodilation [18, 24]. The olfactory bulb receives cholinergic neural inputs originating in the HDB in the basal forebrain [5, 35]. Both in olfactory bulb and HDB cholinergic neurons, mRNA expression of both the α4 and β2 nAChR subunits has been identified in rats [21, 52, 53]. Thus, the nicotine-induced potentiation of olfactory sensory processing in the olfactory bulb could be due to activation of α4β2-like neuronal nAChRs in the olfactory bulb and/or in HDB cholinergic neurons.

### Aging

The investigation of nicotine-induced potentiation of olfactory bulb blood flow responses induced by olfactory nerve stimulation in adult rats (4–8 months old) described above was then extended to animals of older age [54]. In old rats of 24–27 months (approximately 2 years old), olfactory nerve stimulation produces vasodilation in the olfactory bulb. However, the nicotine-induced potentiation of olfactory bulb vasodilation due to α4β2-like nAChR activation decreased considerably in old rats (Fig. 1B). In contrast, the olfactory bulb vasodilatory response to hypercapnic stimulation, indicating the vasodilatory ability of the olfactory bulb, was considerably greater than its response to olfactory nerve stimulation. Thus, we consider that with age, the olfactory bulb blood vessels maintain their vasodilatory ability but with lower reactivity to nicotine. This suggests a decline in α4β2-like nAChR function involving the nicotine-induced potentiation of olfactory bulb vasodilation in old rats.

In old rats of 24–27 months, electrical stimulation of unilateral olfactory nerve increased blood flow in the olfactory bulb ipsilateral to the stimulus without changes in mean arterial pressure [54]. The spatiotemporal blood flow response characteristics and the current and frequency dependence of prompt vasodilation of the olfactory bulb were identical to those observed in adult rats [47]. This is consistent with Kass et al. [55], who described that the odor-evoked synaptic output from the olfactory sensory neurons to the olfactory bulb glomeruli is relatively stable in anesthetized mice of 6–24 months old.

## Comparison of aging effects on the blood flow responses in the neocortex and the olfactory bulb

As described above, the α4β2-like nAChR-mediated vasodilation in the neocortex induced by nicotine injection is relatively well maintained in old rats (23–26 months old) but markedly declines in very old rats (32–36 months old) [18, 24]. On the other hand, the α4β2-like nAChR-mediated potentiation of olfactory bulb vasodilation induced by nicotine injection is reduced in old rats (24–27 months old) [54]. Accordingly, we assume that the age-related impairment of α4β2-like nAChR function may affect the olfactory bulb earlier than the neocortex.

In rat brains, α4 and β2 mRNA levels decrease from 7 to 29 months of age, further decreasing at 32 months. This tendency is relatively constant across in different areas of the brain including neocortex, although olfactory bub is not analyzed [56]. Similarly, in human neocortex, decreases in α4 and β2 mRNA levels as well as α4β2 nAChR availability have been reported with normal aging [57, 58] and AD [59]. The diminished olfactory bulb blood flow potentiation effects of nicotine in old rats as well as diminished nicotine-induced neocortical vasodilation in very old rats may be due to the decline in α4β2 nAChRs in the brain. Further studies comparing the aging effects on neocortex and olfactory bulb, regarding α4β2 nAChR availability, are needed.

Age-related impairment of the regulation of blood flow in the olfactory bulb and neocortex mediated by α4β2-like nAChRs may not be comparable to the baseline regional cerebral blood flow, at least in rodents. This is because the baseline regional blood flow in the neocortex and olfactory bulb is not significantly different in 12-, 24-, and 34-month-old conscious rats when measured using the [^14^C]-iodoantipyrine method [60]. Similarly, using microsphere methods, the baseline regional blood flow in the olfactory bulb has been shown to remain unchanged in 6- and 24-month-old conscious rats [61]. In contrast, in the human brain, resting (baseline) gray matter cerebral blood flow, including that in the frontal regions, is decreased between the ages of 40 and 100 years, as measured using the ^133^X inhalation method [62]. Similar age-related reductions in gray matter cerebral blood flow have been reported by studies that used positron emission tomography [63] and pseudocontinuous arterial spin labeling (pCASL) MRI [64].

## Clinical significance

This review emphasized the crucial role of α4β2-like nAChRs in the brain in neocortical vasodilation and potentiation of olfactory bulb vasodilation in response to olfactory stimulation. A considerable decrease of these α4β2-like nAChR functions occur in older animals at around 2 years old in the olfactory bulb, and later at around 3 years old in the neocortex. The age-related impairment of nicotinic cholinergic regulation of cerebral blood flow in the neocortex and olfactory bulb may explain the deterioration of olfactory and cognitive function in older people [65, 66]. The earlier decline of nAChR function in the olfactory bulb than in the neocortex may explain why the olfactory dysfunction is the earliest symptoms of AD [2, 3]. Human studies have described the relationship between olfaction, cognitive function, regional cerebral blood flow, and the nicotinic cholinergic system. Pilot studies among community-dwelling older adults have shown that older individuals with a higher olfactory identification threshold for rose odor exhibited a greater decline in cognitive function, particularly in attention and discrimination abilities [67, 68]. Attention and discrimination abilities are related to the basal forebrain cholinergic system [69, 70] and undergo early impairment related to AD [71, 72]. Cortical nAChRs, as assessed in vivo using ^11^C-nicotine binding in patients with mild AD, are robustly associated with attention cognitive function [73]. Moreover, the olfactory identification score was negatively correlated with regional cerebral blood flow in several brain areas including the bilateral frontal pole, in patients with mild cognitive impairment (MCI) and AD [74].

Since the α4β2-like nAChRs in the brain decline with age as well as in AD, activation of the nAChRs involved in cortical vasodilatation or potentiation of olfactory bulb vasodilation could be beneficial for older people and AD patients. To this end, administering nicotinic receptor agonists or physical therapies such as somatosensory stimulation and walking, known to activate basal forebrain cholinergic system in both adult and old rats [75–80], may have therapeutic values. Furthermore, the increased cerebral blood flow in the neocortex and olfactory bulb induced by α4β2-like nAChR activation could improve oxygen and glucose delivering to those brain areas, and those sufficient nourishments appear to be beneficial for neuronal protection and maintaining cognitive function and olfaction.

## Data Availability

Not applicable.

## Abbreviations

AD: Alzheimer’s disease
HDB: Horizontal limb of the diagonal band of Broca
MCI: Mild cognitive impairment
MS: Medial septal nucleus
NBM: Nucleus basalis of Meynert
SI: Substantia innominate
VDB: Ventral limb of the diagonal band of Broca
nAChR: Nicotinic acetylcholine receptor
